# Metabolomic changes of the multi (-AGC-) kinase inhibitor AT13148 in cells, mice and patients are associated with NOS regulation

**DOI:** 10.1007/s11306-020-01676-0

**Published:** 2020-04-13

**Authors:** Akos Pal, Yasmin Asad, Ruth Ruddle, Alan T. Henley, Karen Swales, Shaun Decordova, Suzanne A . Eccles, Ian Collins, Michelle D. Garrett, Johann De Bono, Udai Banerji, Florence I. Raynaud

**Affiliations:** 1 Division of Cancer Therapeutics, The Institute of Cancer Research, London, SW7 3RP UK; 2 Drug Development Unit, The Royal Marsden NHS Foundation Trust, Sutton, UK; 3 School of Biosciences, University of Kent, Canterbury, UK

**Keywords:** AT13148, Targeted metabolomics, NOS, Hypotension, ADMA

## Abstract

**Introduction:**

To generate biomarkers of target engagement or predictive response for multi-target drugs is challenging. One such compound is the multi-AGC kinase inhibitor AT13148. Metabolic signatures of selective signal transduction inhibitors identified in preclinical models have previously been confirmed in early clinical studies. This study explores whether metabolic signatures could be used as biomarkers for the multi-AGC kinase inhibitor AT13148.

**Objectives:**

To identify metabolomic changes of biomarkers of multi-AGC kinase inhibitor AT13148 in cells, xenograft / mouse models and in patients in a Phase I clinical study.

**Methods:**

HILIC LC–MS/MS methods and Biocrates AbsoluteIDQ™ p180 kit were used for targeted metabolomics; followed by multivariate data analysis in SIMCA and statistical analysis in Graphpad. Metaboanalyst and String were used for network analysis.

**Results:**

BT474 and PC3 cells treated with AT13148 affected metabolites which are in a gene protein metabolite network associated with Nitric oxide synthases (NOS). In mice bearing the human tumour xenografts BT474 and PC3, AT13148 treatment did not produce a common robust tumour specific metabolite change. However, AT13148 treatment of non-tumour bearing mice revealed 45 metabolites that were different from non-treated mice. These changes were also observed in patients at doses where biomarker modulation was observed. Further network analysis of these metabolites indicated enrichment for genes associated with the NOS pathway. The impact of AT13148 on the metabolite changes and the involvement of NOS-AT13148- Asymmetric dimethylarginine (ADMA) interaction were consistent with hypotension observed in patients in higher dose cohorts (160-300 mg).

**Conclusion:**

AT13148 affects metabolites associated with NOS in cells, mice and patients which is consistent with the clinical dose-limiting hypotension.

**Electronic supplementary material:**

The online version of this article (10.1007/s11306-020-01676-0) contains supplementary material, which is available to authorized users.

## Introduction

The study of small molecule metabolites utilizing metabolomics is a flourishing technique in the biological milieu. It provides insight into normal physiology and pathomechanisms, and tools to characterise phenotypes. In the context of drug discovery and development, metabolites provide accessible biomarkers (Ang et al. 2017, 2016; Griffiths et al. 2010).This is particularly important where the availability of human tumour biopsies is limited and the use of surrogate tissues, e.g. plasma, is essential to assess target engagement. Metabolite changes can be evaluated in models of increasing complexity, i.e. tumour cells, mouse xenograft studies and clinical samples. In addition to tumour mediated effects, assessment of the compound non-tumour related effects has the potential to (i) identify biomarkers, (ii) gain better understanding of the mechanism of action and (iii) predict potential side effects earlier in the drug development process before the clinical phases. We have previously demonstrated that metabolomic signatures established in preclinical studies can be used clinically as biomarkers of target engagement and/or predictors of tumour and compound specific responses (Ang et al. 2017, 2016). In these studies, a workflow to generate tumour specific and target related metabolomic signatures was successfully applied. Initially, metabolomic changes in plasma induced by different pathway inhibitors in preclinical studies from tumour and in non-tumour bearing mice were measured. These preclinical data sets were used to generate target and tumour specific metabolomic signatures which were assessed later with plasma samples from patients in a Phase I clinical trial (Ang et al. 2017, 2016; Sarker et al. 2015; Yang et al. 2019).

AT13148 is an orally available, potent broad spectrum AGC kinase inhibitor. It is an ATP competitive inhibitor of PKA (3 nM), ROCK1 (6 nM), ROCK2 (4 nM), p70S6K (8 nM), PRK1 (16 nM), AKT1 (38 nM), AKT3 (50 nM), SGK3 (63 nM) and RSK1 (85 nM) (Yap et al. 2012). In cancer cells, AT13148 blocks phosphorylation of AKT, p70S6K, PKA, ROCK, and SGK substrates and induces apoptosis; it shows in vivo anti-tumour efficacy in HER2-positive, PIK3CA-mutant BT474 breast, PTEN-deficient PC3 human prostate cancer, and PTEN-deficient MES-SA uterine human xenografts in mice (Yap et al. 2012). A dose escalation Phase I clinical trial of AT13148 was undertaken in patients with advanced solid tumours.

In this study, we tested the effect of AT13148 on cellular metabolites, plasma metabolites in mice bearing human tumour xenografts sensitive to AT13148 and in non-tumour bearing mice post treatment. Finally, we compared these preclinical results to metabolite changes observed in the Phase 1 dose escalation study carried out at the Royal Marsden Hospital.

## Materials and methods

### Cellular screen

BT474 (ATCC lot #3,272,826, 13/02/03) and PC3 (ATCC lot #61,573,377 07/07/2015), were profiled and authenticated in house. Cell lines were analyzed by short tandem repeat (STR) profiling. Polymorphic STR loci were amplified using a PCR primer set. The PCR product (each locus was labelled with a different fluorophore) was analysed simultaneously with size standards by using an automated fluorescent detection technique. The number of repeats at 7–10 different loci defines the STR profile and was cross-referenced with online databases to confirm authenticity. All cell lines showed 100% match and were negative for mycoplasma.

BT474 cells were maintained in Dulbecco’s Modified Eagle Medium (DMEM)/F-12 supplemented with 10% fetal bovine serum and insulin 10 µg/mL. PC3 cells were cultured in Dulbecco’s Modified Eagle Medium (DMEM) supplemented with 10% fetal bovine serum. Both cell lines were kept at 37 °C, 5% CO2 in a humidified incubator and media were changed in every 2–3 days and cells were allowed to reach 80% confluency before passages.

The cells were incubated with 1 µM AT13148 (to mimic in vivo exposure) and the same volume of vehicle control (DMSO < 0.1% (v/v).) for 24 h in T25 flasks. Metabolites were extracted with methanol at − 80 °C and samples were dried under nitrogen to evaporate methanol before LC–MS/MS analysis.

The selected metabolites and associated proteins were analysed to map their interactions in different pathways and networks (https://www.metaboanalyst.ca and https://string-db.org/).

### Measurement of cellular ADMA

The effect of AT13148 on the intracellular level of Asymmetric dimethylarginine (ADMA) was monitored in BT474 cells treated with AT13148 (1 µM, for 6 and 24 h compared with their DMSO controls from the same experiment; 3 flasks/condition, DMSO < 0.1% (v/v)). The samples were analysed with a short HILIC LC–MS/MS method, which includes ADMA, (adapted from a method by Shina et al. 2011).

### Measurement of nitric oxide activity

NOS activity was measured following treatment of BT474 cells with AT13148 (1 µM, 6 and 24 h; 3 flasks/condition; DMSO < 0.1% (v/v)); the cells were washed with PBS and analysed for NOS activity by a colorimetric method on a Biotech Synergy HT plate reader according to the manufacturer`s instructions (Abcam, Cambridge, UK).

### Preclinical mouse studies

BT474 (ATCC lot #3,272,826, 13/02/03) and PC3 (ATCC lot #61,573,377 07/07/2015) were profiled and authenticated in house (2015) as described above.

For pharmacodynamics and metabolomics experiments 2 million cells were injected in PBS subcutaneously bilaterally into the flanks of female NCr athymic mice 6–8 weeks of age, bred in-house. During the experiment, food pellets (Certified Rodent Diet 5002, Labdiet, Indianapolis, Indiana, USA) and water were available ad libitum. Dosing of the animals was undertaken synchronously under sterile conditions in the same experiment when tumours were well-established and approximately 8–10 mm in diameter. A single dose of 40 mg/kg AT13148 was administered p.o. in 10% DMSO, 1% TWEEN 20, 89% saline. Control animals received an equivalent volume of vehicle.

To observe non-tumour specific metabolic changes, non-tumour bearing mice were treated the same way as the tumour-bearing mice.

Blood samples were collected at 2, 6 and 24 h after drug administration. 3–4 mice were used for each time point per treatment.

The blood samples were centrifuged at 13,000 rpm for 2 min and the plasma transferred onto dry ice; the entire process from collection to storage in dry ice took less than 5 min per sample. Plasma samples were stored at -80 °C until further LC–MS/MS analysis.

### Phase I clinical trial of AT13148

Plasma samples for metabolomic analysis were obtained from 25 patients with advanced tumours across 5 cohorts as part of a dose escalation Phase I study with AT13148 (Clinical trial information: NCT01585701. Demographic profile of the trial is summarized in Supplementary Table 1). AT13148 was administered according to a weekly schedule once per day on days 1, 3 and 5. One treatment cycle was 28 days. Following a pre-dose sample for baseline levels, Cycle 1 (Day 7 to 4) blood samples (2 mL) were taken. Blood (with sodium heparin as anticoagulant) was centrifuged (1500 g for 15 min at 4 °C); the plasma was separated then stored at –80 °C until further LC–MS/MS analysis. Metabolomics analysis was carried out on samples from patients dosed with 40 mg, 80 mg, 160 mg, 240 mg or 300 mg of AT13148 daily and samples were collected pre-dose, 6, 12 and 24 h post-dose in Cycle 1.

**Table 1:**
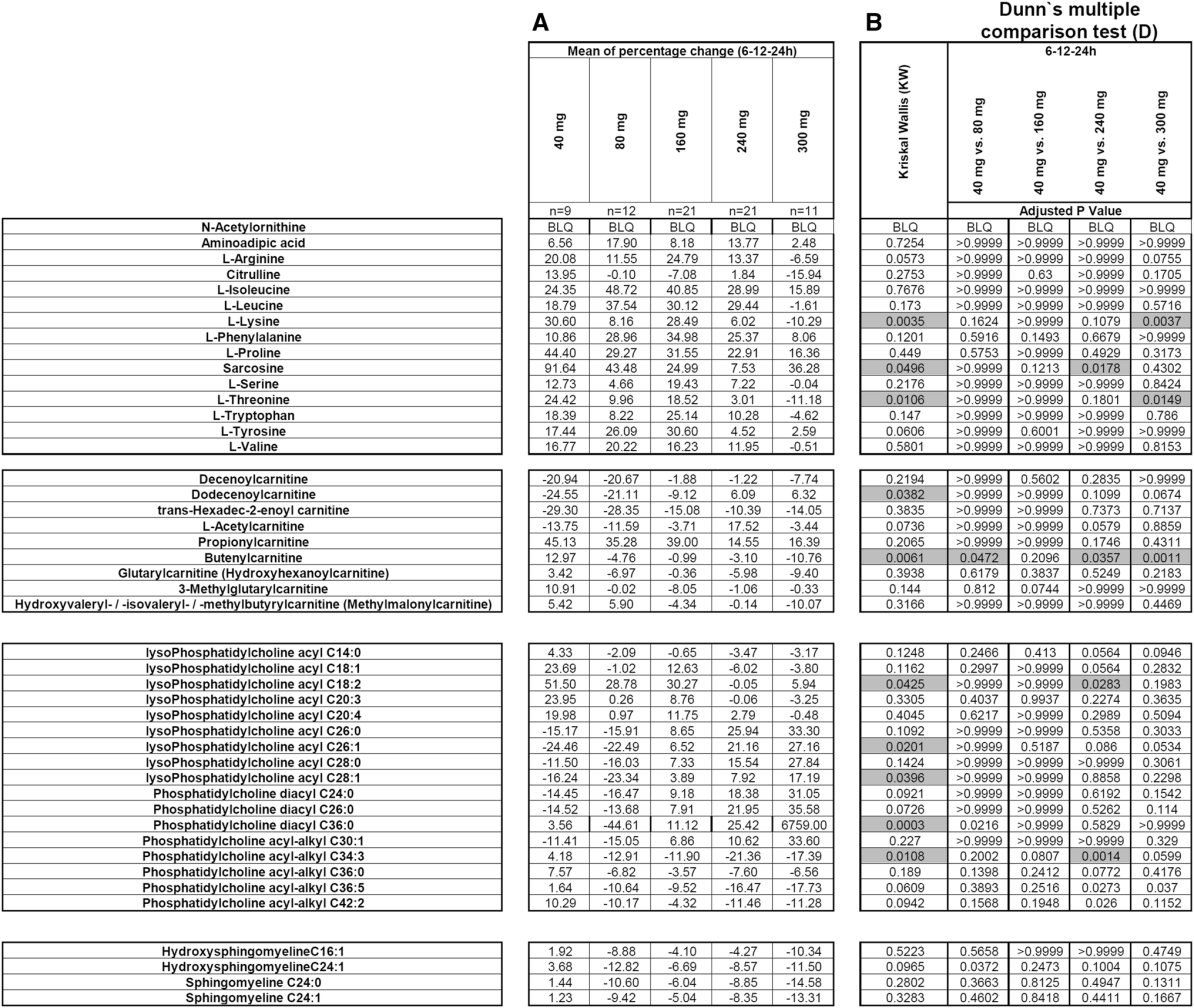
Mean percentage changes compared to predose level (6–12-24 h)

Kruskal–Wallis and Dunn`s test per doses (6–12-24 h); (Ac-Orn excluded, due to BLQ). The 10 statistically significant (Kruskal–Wallis test, greyed) metabolites in samples from patients dosed with 80,160,240 and 300 mg and compared to sub-therapeutic 40 mg

### Analysis of Clinical PD Biomarkers

GSK3β assay was performed on platelet rich plasma (Banerji et al. 2018) using assays validated to Good Clinical Practice standards on the MesoScale Discovery (MSD®). To monitor the phosphorylation status of GSK3β the samples were added to the plate followed by a solution containing the MSD SULFO-TAGTM labelled detection antibody for GSK3β.The plate was then analysed on the MSD SECTORTM Imager 6000 (Yap et al. 2014). The levels of pSer9 GSK3β, represented as a percent of the pre-treatment levels, were normalised to the levels of total GSK3β.

Myosin regulatory light chain 2 (MLC-2) levels of phosphorylated Ser19 and total MLC-2 were measured in lysates of platelets, isolated from blood by centrifugation, by Western blotting using fluorescent tagged IgG secondary antibodies and GAPDH as a loading control. The assay was validated by the ICR Clinical PD Biomarker group for use in clinical trials including low, medium and high Quality Controls. Fluorescent detection by LI-COR Odyssey Fc system and densitometric analysis of the images was performed using Image Studio (LI-COR).

### LC–MS/MS Targeted metabolomics analysis of cell and plasma samples

Cells: PC3 and BT474 cell extracts were analysed using targeted non-quantitative LC–MS/MS with HILIC chromatography and a positive/negative ion–switching method MS (adapted method of Yuan et al. 2012, all monitored metabolites are listed in Supplementary material). Briefly, previously dried extracts were reconstituted in acetonitrile–water (1:1) and analysed by LC–MS/MS on a QTrap6500 mass spectrometer (AB Sciex, Warrington, UK). Chromatography was performed using a Shimadzu Nexera UPLC system on an Amide XBridge HPLC column (3.5 μm; 4.6 mm inner diameter (i.d.) × 100 mm length). A gradient mobile phase with acetonitrile and 20 mM ammonium acetate, buffered at pH 9, was used with flow rate of 0.3 mL/min and a run time of 23 min (Yuan et al. 2012). A second shorter HILIC method (10 min) for optimisation of arginines (including ADMA) was carried out on a similar column and conditions using a gradient mobile phase containing 0.5% acetic acid with 0.025% TFA (Shina et al. 2011). Analyst® software was used for data acquisition.

Plasma: mouse and patient plasma samples were analysed using targeted, quantitative metabolomic analysis by electrospray ionization tandem MS using the AbsoluteIDQ™ p180 kit (Biocrates Life Sciences AG, Innsbruck, Austria). Samples were derivatised using phenylisothiocyanate (PITC). Metabolites were extracted and analysed according to the manufacturer`s instructions. Preclinical and clinical plasma samples were anonymized and randomized and analyses were carried out on a Waters Acquity H-class UPLC coupled to a Xevo TQ-S triple-quadrupole MS/MS System (Waters Corporation, Manchester, UK). Quantification of the metabolites of each biological sample was achieved by reference to appropriate stable isotope internal standards. The method follows the United States Food and Drug Administration Guidelines ‘Guidance for Industry—Bioanalytical Method Validation (May 2001)’, providing proof of reproducibility within a given error range.

### Data analysis

MultiQuant® (version 3.0.3) was used for processing cellular metabolites to monitor their changes compared to controls (after normalization by the total sum of monitored analyte area). To derive metabolite concentrations in mouse and human plasma the analytical process was performed using MassLynx™ and TargetLynx™ (Waters corporation Manchester UK) and the MetIDQ™ software package (Biocrates Life Sciences, Innsbruck, Austria.)

Multivariate analysis was performed on cellular and plasma samples by SIMCA v.15 software (MKS Umetrics AB, Sweden); to determine metabolite features that were differentially expressed between defined sample groups. Metabolites responsible for differences were identified using OPLS-DA with a threshold of variable importance in the projection (VIP) value ≥ 1 and minimum 0.5 p_corr_ absolute value on S-plot.

For the relevant cell or plasma metabolites the changes relative to control concentration (solvent, vehicle or sub-therapeutic dose) were used to generate heat maps. We tested the metabolomics signature identified in non-tumour bearing mice in samples from Phase I clinical trial patients.

In clinical plasma samples, changes relative to pre-treatment baseline levels were calculated for each patient across all time points for each metabolite and normalised to the sub-therapeutic dose, 40 mg, to eliminate the food and time of day effects.

The statistical significance of the differences were determined using Mann–Whitney (cell data) or Kruskall-Wallis followed by Dunn`s multiple comparison post hoc (clinical data) tests (Graphpad Prism v7 and v8, California, USA), and values < 0.05 were considered statistically significant.

The network diagrams were constructed using MetaboAnalyst 3.0 (gene-metabolites) and String (proteins) (https://www.metaboanalyst.ca and https://string-db.org/ retrospectively).

## Results and discussion

### Cell experiments

**Fig. 1:**
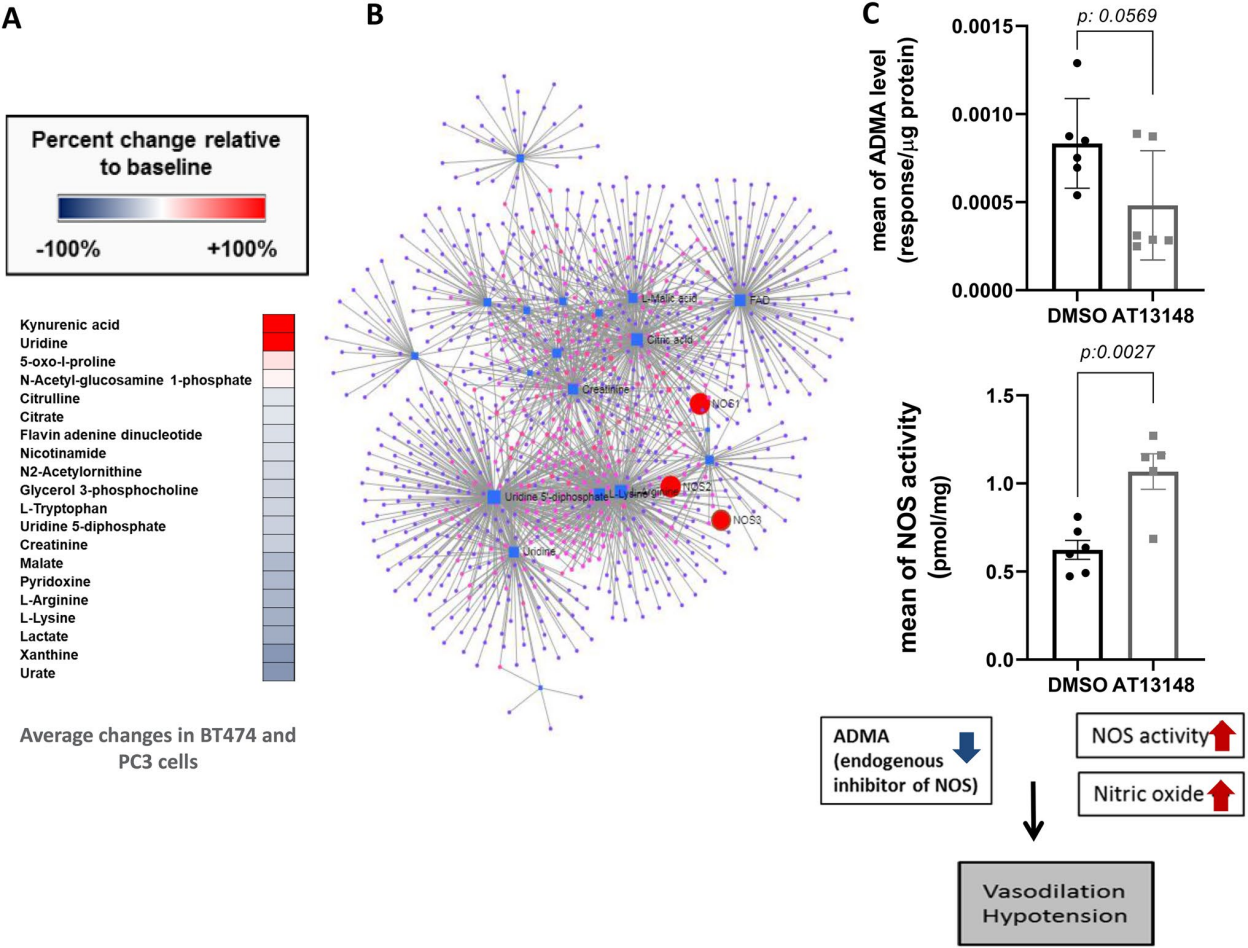
Summary of in vitro cellular work **a** Average changes of selected 20 metabolites of BT474 and PC3 cells, **b** Metabolite- Gene network based on 20 selected metabolites modulated by AT13148 in cells (including NOS1, NOS2, NOS3). **c** Averages of 6 and 24 h changes (± SEM) of intracellular level of Asymmetric dimethylarginine (ADMA) decreased in the presence of AT13148 and induced NOS activity in BT474 cells

Following treatment of the human breast carcinoma cells BT474 and the PC3 prostate carcinoma cells with 1 µM AT13148, 20 metabolites were selected based on the following criteria: VIP > 1 and abs p_corr_ > 0.5. The average percentage changes relative to control are presented in Fig. 1a. These included Kynurenic acid, Uridine, 5-oxo-l-proline, N-Acetyl-glucosamine 1-phosphate, Citrulline, Citrate, Flavin adenine dinucleotide, Nicotinamide, N2-Acetylornithine, Glycerol 3-phosphocholine, L-Tryptophan, Uridine 5-diphosphate, Creatinine, Malate, Pyridoxine, L-Arginine, L-Lysine, Lactate, Xanthine and Urate. Importing these metabolites into Metaboanalyst, (Fig. 1b), shows that these metabolites are associated with NOS. We therefore decided to assess NOS activity in BT474 cells treated with AT13148, and measure ADMA, which is a naturally occurring endogenous inhibitor of nitric oxide synthases (Latika Sibal et al. 2010). We found that AT13148 decreases Asymmetric dimethylarginine (ADMA) levels and increases NOS activity compared with controls (Combined 6 and 24 values are on Fig. 1c).

### Preclinical and clinical plasma samples

#### Preclinical tumour specific signature

The effect of AT13148 on mouse plasma metabolites from PKPD experiments in the sensitive tumour xenograft models BT474 and PC3 was assessed. We sought to identify metabolites that were different in tumour bearing mice compared with controls and where treatment with AT13148 40 mg/kg reversed the effect. After evaluation of the selected metabolite changes, we were not able to generate a robust AT13148 metabolomics signature (based on the OPLS-DA selection criteria) that was reversible and specifically associated with tumour-bearing mice that responded to AT13148 (Yap et al. 2012) and not present in non-tumour bearing animals. This could be due to the limited number of sensitive tumours used for this analysis and to the fact that AT13148 has multiple targets.

#### Preclinical non-tumour specific signature

**Fig. 2:**
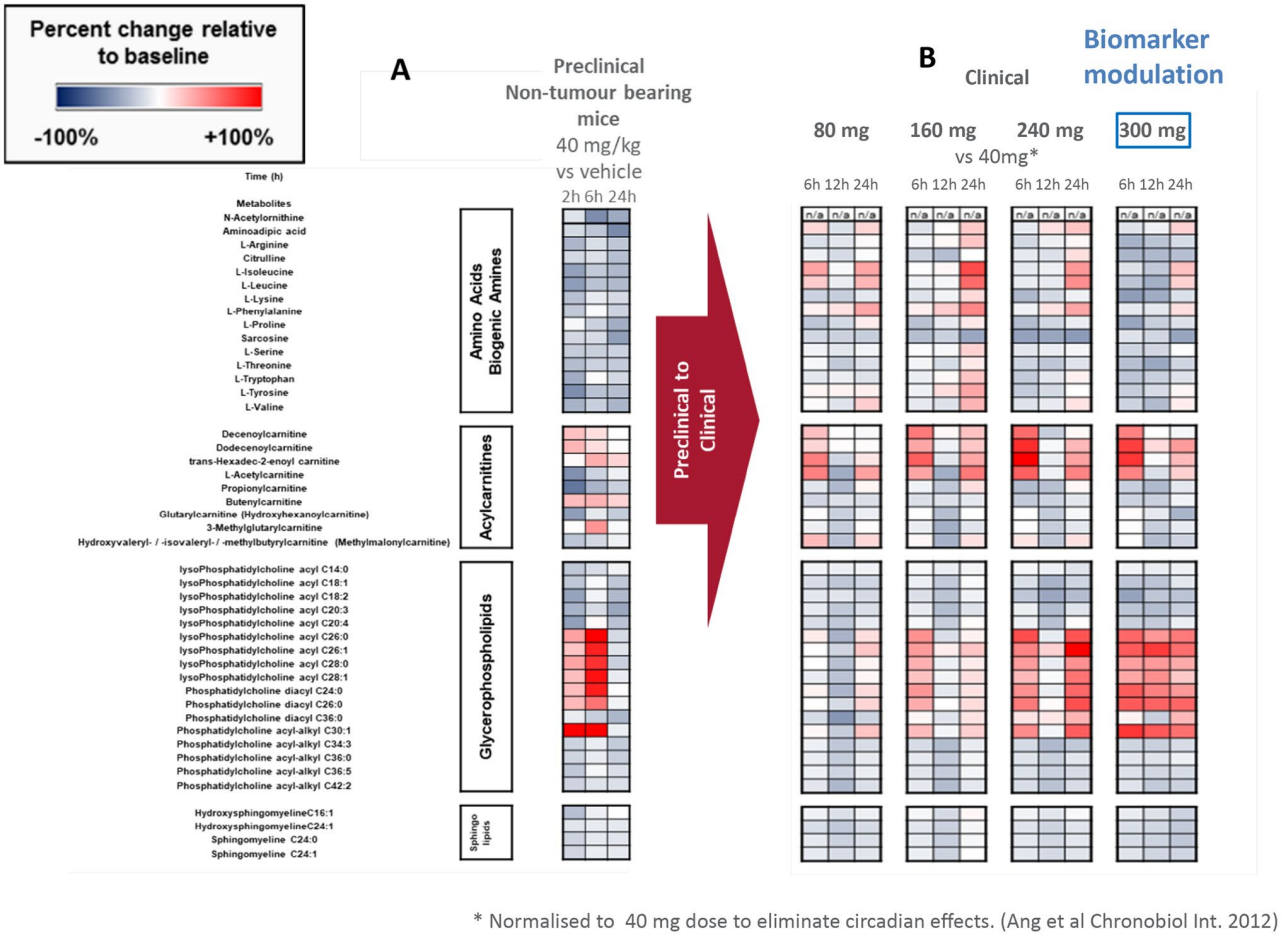
Heatmaps illustrating the changes of **a** the preclinically selected metabolites in (non-tumour bearing) mice and **b** preclinical signature translated to the clinic

In parallel, with the aim to utilize the non-tumour bearing mouse model as a potential tool to predict non-tumour specific effects of single dose AT13148, we analysed the preclinical plasma samples derived from non-tumour bearing mice. We compared metabolite changes in plasma from mice treated with 40 mg/kg of AT13148 and their vehicle control group. In OPLS-DA analysis, we identified 45 metabolites with a minimum VIP value of 1 and minimum absolute value of p_corr_ 0.5 on S-Plot (Fig. 2a; list of metabolites including VIP and p_corr_ scores are listed in the supplementary data). These include amino acids, biogenic amines, acylcarnitines and glycerophospholipids. 36 out of 45 metabolites were available in the HMDB database for further network analysis (Supplementary figure S1b).

#### Clinical translation of the signature based on preclinical non-tumour bearing mouse models

Following normalisation to the 40 mg dose to remove the time of day variation in plasma metabolites, 44 out of the 45 metabolites identified in mice following AT13148 treatment were also affected in patients. Of note is that these effects were only observed at doses of 160 mg and above (Fig. 2a and b). The observation that these metabolic effects occurred at doses where the biomarkers are modulated confirms that they are associated with AT13148.

**Fig. 3:**
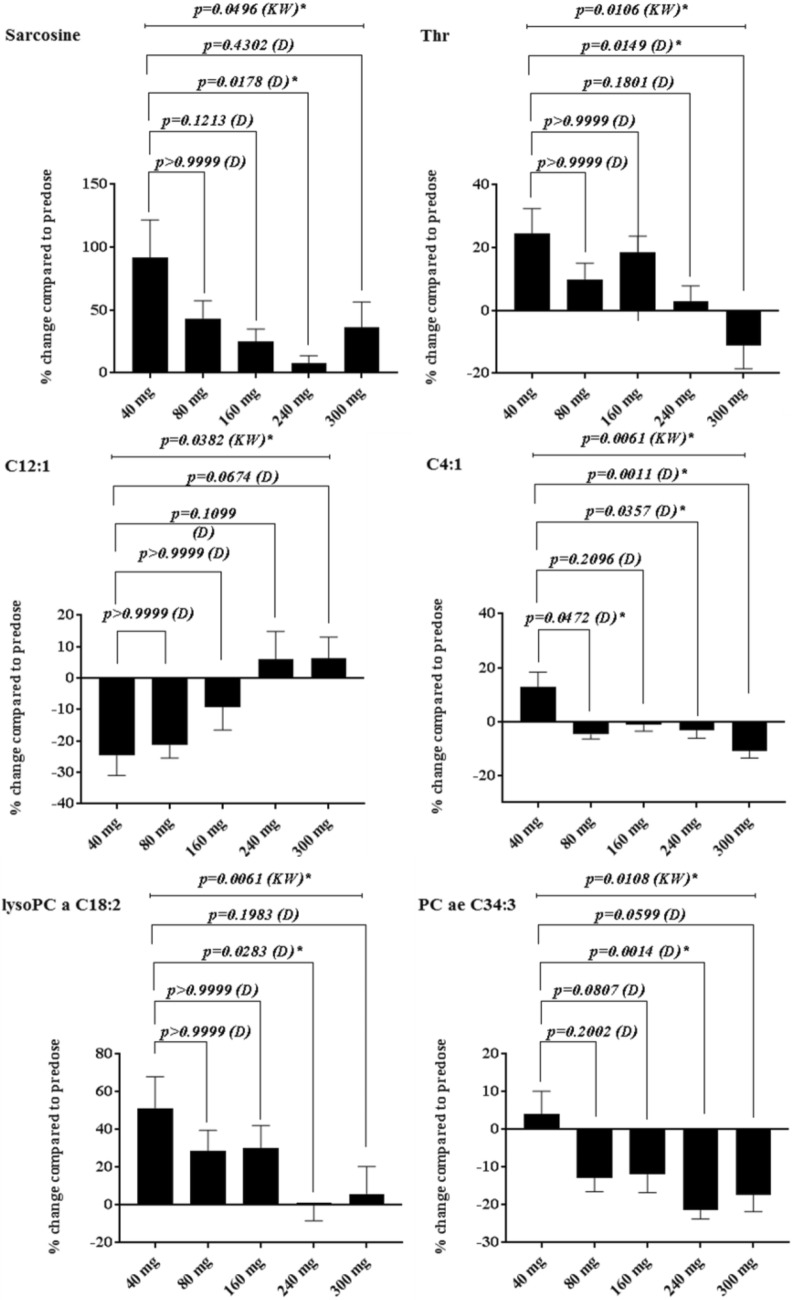
Representative examples of statistically significant metabolite changes in patient samples across clinical cohorts. Sarcosine, Butenylcarnitine (C4:1), L-Threonine(Thr), lysoPhosphatidylcholine acyl C18:2(lysoPC a C18:2), Phosphatidylcholine acyl-alkyl C34:3 (PC ae C34:3) Mean (± SEM) of percentage changes compared to predose level, KW: Kruskal–Wallis, D: Dunn’s test

The preclinical signature shows the highest correlation with the clinical signature from patients at 300 mg where on-target biomarker modulation was also observed (Fig. 2 and Supplementary figure S2). ROCK and AKT associated inhibition was also confirmed by biomarker changes (MLC2 Supplementary figure S3 and GSK3β Figure S4) in the clinical trial at doses of 160, 240 and 300 mg. We analysed the clinical data set further to investigate statistically significant metabolite dose responses compared to the sub-therapeutic 40 mg-samples. Despite the small size of the clinical study, we identified the following 10 metabolites (~ 20% of all metabolites measured) Butenylcarnitine (C4:1), Dodecenoylcarnitine (C12:1), L-Lysine (Lys), L-Threonine (Thr), Sarcosine, lysoPhosphatidylcholine acyl C18:2 (lysoPC a C18:2), lysoPhosphatidylcholine acyl C26:1 (lysoPC a C26:1), lysoPhosphatidylcholine acyl C28:1 (lysoPC a C28:1), Phosphatidylcholine diacyl C36:0 (PC aa C36:0), Phosphatidylcholine acyl-alkyl C34:3 (PC ae C34:3) from the preclinical signature which showed statistically significant dose response effects compared with their sub-therapeutic levels. Table 1 shows the results of the statistical analysis and the 10 significantly changed metabolites when applying the preclinical signature to clinical samples. Figure 3 shows representative examples of the dose dependent effects of AT13148 on selected metabolites (combined values of 6–12-24 h).

Further statistical analysis of all detectable clinical metabolites and their dose responses in the 5 clinical cohorts showed 33 metabolites significantly affected by AT13148 (Supplementary data).

One of the most robust changes was the decreased level of Asymmetric dimethylarginine (ADMA) (Kruskal–Wallis p = 0.0044; Supplementary figure S4) (Tran et al. 2003). Application of the network explorer option in Metaboanalyst on the identified 33 significant metabolites showed that NOS enzymes were captured in the constructed network (Supplementary figure S1c).

**Fig. 4:**
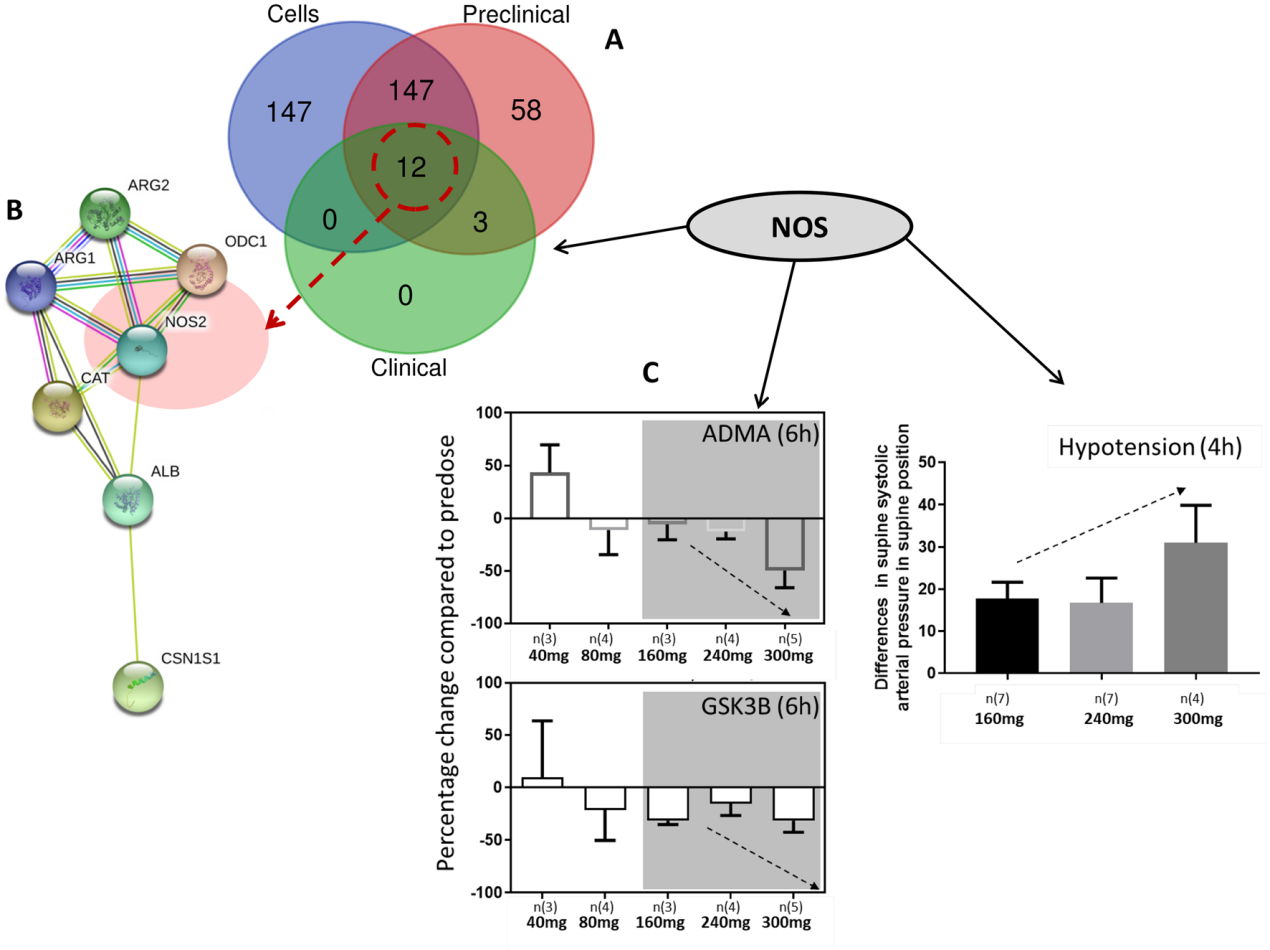
Summary of NOS, ADMA, AKT and hypotension associated results and observations. **a** Venn diagram of genes selected from cells, preclinical and clinical networks **b** Protein network of the common genes in String includes: Nitric oxide synthase (NOS2), Ornithine decarboxylase (ODC1), Catalase (CAT), Alpha-S1-casein (CSN1S1), Arginase-1 (ARG1), Arginase-2 (ARG2), Serum albumin (ALB). **c** Clinically observed, mean (± SEM), dose dependent decrease of Asymmetric dimethylarginine (ADMA) (6 h) and pSer19: Total GSK3β (6 h) resulting in increasing level of hypotension(4 h) (differences increased in systolic arterial pressure in supine position)

Finally, we assessed the common genes from the gene-metabolites networks derived from cells, the preclinical signature and the clinical signature. Genes with a minimum of 2 degree interaction were selected. Twelve common genes (Fig. 4a) were identified in all three networks. Based on this information we generated a protein network in String; 7 out of 12 proteins showed direct interaction; they were also linked to nitric oxide synthase (NOS2) (Fig. 4b). NOS enzymes are controlled by multiple co- and post-translational lipid modifications via endomembrane specific regulation by several classes of signalling molecules, including phosphatidylinositol 3,4,5-trisphosphate (Sato et al. 2003), Ras (Bivona and Philips 2003), and G proteins (Stow and Heimann 1998). Endothelial NOS (NOS3) activity is also regulated by phosphorylation and by protein–protein interactions (Fulton et al. 2004). ROCK negatively regulates the function of endothelial nitric oxide synthase eNOS (NOS3) by inhibiting AKT phosphorylation of eNOS on Ser-1177 leading to decreased NO production (Ming et al. 2002). Inhibition of AKT was observed in our clinical study with a concentration dependent inhibition of the ratio of phosphorylated over total GSK3β (Fig. 4c). The impact of AT13148 on the metabolite changes, including a significant and dose-dependent decrease of Asymmetric dimethylarginine (ADMA) and the involvement of NOS, was associated with hypotension observed in patients at the higher doses of 160, 240 and 300 mg (Fig. 4c). The direct interaction between ADMA-NOS and AT13148 is consistent with our measurements of NOS activity and Asymmetric dimethylarginine levels in BT474 cells, (Fig. 1c) and the vascular smooth muscle contraction, hypotension, and tachycardia seen in mice treated with AT13148 (Yap et al. 2012). Asymmetric dimethylarginine (ADMA) is a naturally occurring endogenous inhibitor of nitric oxide synthases (Latika Sibal et al. 2010, Tran et al. 2003). The decreased level of ADMA may have been caused by inhibition of protein arginine methyltransferase type 1 (PRMT1) which is responsible for the formation of NG-monomethyl-L-arginine (LNMMA) and NG, NG-dimethyl-L-arginine (ADMA) (Latika Sibal et al. 2010, Tran et al. 2003). In addition, dimethylarginine, dimethylaminohydrolases (DDAHs), enzymes removing methylarginines, such as ADMA, could have some influence on the AT13148 associated metabolic changes/pathways observed; DDAH1 is involved in Ras-pathway regulation therefore indirectly could have an impact on NOS activity, while DDAH2 binds directly to PKA which can also be associated with the increased lipid changes observed in plasma samples (Leiper and Vallance 2006). Our results suggest that the underlying mechanism of the clinically observed hypotension (in systolic arterial pressure in the supine position) triggered by AT13148 might be an increased ADMA-sensitive NOS activation resulting in elevated production of the endogenous vasodilator NO. It is consistent with previous observations with the ROCK inhibitor fasudil which is used in Japan for cerebral vasospasm (Murakami et al. 2019).

We observed significant increases in lipids both in non-tumour bearing mice and in patients. AT13148 inhibits AKT which has been shown to enhance de novo lipid synthesis in the liver (Yecies et al. 2011). In patients, reduction of GSK3β phosphorylation was observed indicating inhibition of AKT. Although we have not demonstrated a clear inhibition of PKA, AT13148 is also a potent PKA inhibitor. PKA phosphorylates numerous enzymes in lipid signalling including, for example, acetyl CoA carboxylase, which inhibits lipid synthesis and inactivates phospholipase C. This may explain the observed dose dependent increase of glycerophospholipids (lysoPhosphatidylcholines (lysopPCs) and Phosphatidylcholines(PCs) (Sassone-Corsi 2012; Liao et al. 2007; Ming et al. 2002; Pearce et al. 2010).

## Conclusions

This is our third study successfully utilizing targeted LC–MS/MS metabolomics to identify compound related changes in preclinical and clinical samples. Previously, in two selective signal transduction inhibitor (PI3K and MEK) studies, we have demonstrated that targeted LC–MS/MS metabolomics is a powerful approach in cancer biomarker studies and has the capability to establish metabolomic signatures of target engagement and predictive response. Based on preclinical xenograft models, tumour specific metabolite changes were identified upon treatment with inhibitors and successfully applied to the clinic. AT13148 is a pan AGC-kinase inhibitor and we were unable to establish a tumour specific signature as the metabolic effects observed in sensitive tumour cells are also seen in normal tissues. Although this is a Phase I study with a limited number of patients with heterogenous cancers treated at different dose levels, many of the metabolites modulated by AT13148 in patients’ plasma were also affected in mice. Despite the fact that the gene-metabolite-protein combined network analysis relies on pre-existing data bases with their own limitations, cellular, animal data and patient analysis consistently associated the metabolic signature with the NOS pathway. This was further validated with ADMA and NOS measurement.

Our data suggest that AT13148 activates NOS resulting in elevated production of the natural vasodilator NO resulting in the clinical dose-dependent hypotension observed.

## Electronic supplementary material

Below is the link to the electronic supplementary material.

Supplementary file1 (DOCX 13 kb)

Supplementary file2 (XLSX 10 kb)

Supplementary file3 (XLSX 11 kb)
