# Supplementary material for: Metabolomic changes of the multi (-AGC-) kinase inhibitor AT13148 in cells, mice and patients are associated with NOS regulation
Source: Metabolomics. 2020 Apr 13;16(4):50. doi: 10.1007/s11306-020-01676-0 (PMC7154022; doi:10.1007/s11306-020-01676-0)
Supplement: Supplementary file 1 — Supplementary file1 (DOCX 13 kb) [file 11306_2020_1676_MOESM1_ESM.docx]

| **Characteristics** | | | **Male** | **Female** | **Total** |
| --- | --- | --- | --- | --- | --- |
| Gender | | | 25 | 31 | 56 |
| Age (years) | | Median | 58  34-76 | 60  39-76 | 59.5  34-76 |
|  |  | Range |  |  |  |
| Performance status | | 0 | 11  14 | 13  18 | 24  32 |
|  |  | 1 |  |  |  |
| Tumour type | 1. Colorectal 2. Breast 3. Pancreas 4. Bile duct/gall bladder 5. Other | | 18  0  0  1  6 | 9  5  3  2  12 | 27  5  3  3  18 |

Table 1

Demographic profile of patients treated with AT13148.
